# The Influence of Diet on Fertility and the Implications for Public Health Nutrition in the United States

**DOI:** 10.3389/fpubh.2018.00211

**Published:** 2018-07-31

**Authors:** Neelima Panth, Adam Gavarkovs, Martha Tamez, Josiemer Mattei

**Affiliations:** ^1^Department of Social and Behavioral Sciences, Harvard T.H. Chan School of Public Health, Boston, MA, United States; ^2^School of Medicine, Duke University, Durham, NC, United States; ^3^Department of Nutrition, Harvard T.H. Chan School of Public Health, Boston, MA, United States

**Keywords:** diet and fertility, nutrition and fertility, infertility, infertility treatment, obesity and fertility, fertility disparities

## Abstract

Despite growing evidence of the impact of diet on human fertility, few studies have examined the public health implications of this association in the United States (U.S.). This narrative review summarizes current scientific evidence on associations between dietary intake and fertility, discusses challenges in the public health landscape surrounding infertility, and proposes evidence-based recommendations to address these issues. Diets high in unsaturated fats, whole grains, vegetables, and fish have been associated with improved fertility in both women and men. While current evidence on the role of dairy, alcohol, and caffeine is inconsistent, saturated fats, and sugar have been associated with poorer fertility outcomes in women and men. Furthermore, women and men with obesity [body mass index (BMI) ≥ 30 kg/m^2^] have a higher risk of infertility. This risk is extended to women who are underweight (BMI <20 kg/m^2^). Diet and BMI influence outcomes during clinical treatment for infertility. Further, women in the U.S. who belong to an underrepresented minority group, have low income, or have low educational attainment, have significantly higher rates of infertility outcomes as compared to women who are non-Hispanic white, have high income, or have high educational attainment. Given this, it may be prudent to integrate nutrition counseling into both clinical guidelines for infertility as well as national dietary guidelines for individuals of reproductive age. Further studies on diet and reproductive health may enhance our ability to improve existing fertility programs across the U.S. and to deliver tailored care to women and men within at-risk groups.

## Introduction

**Figure 1:**
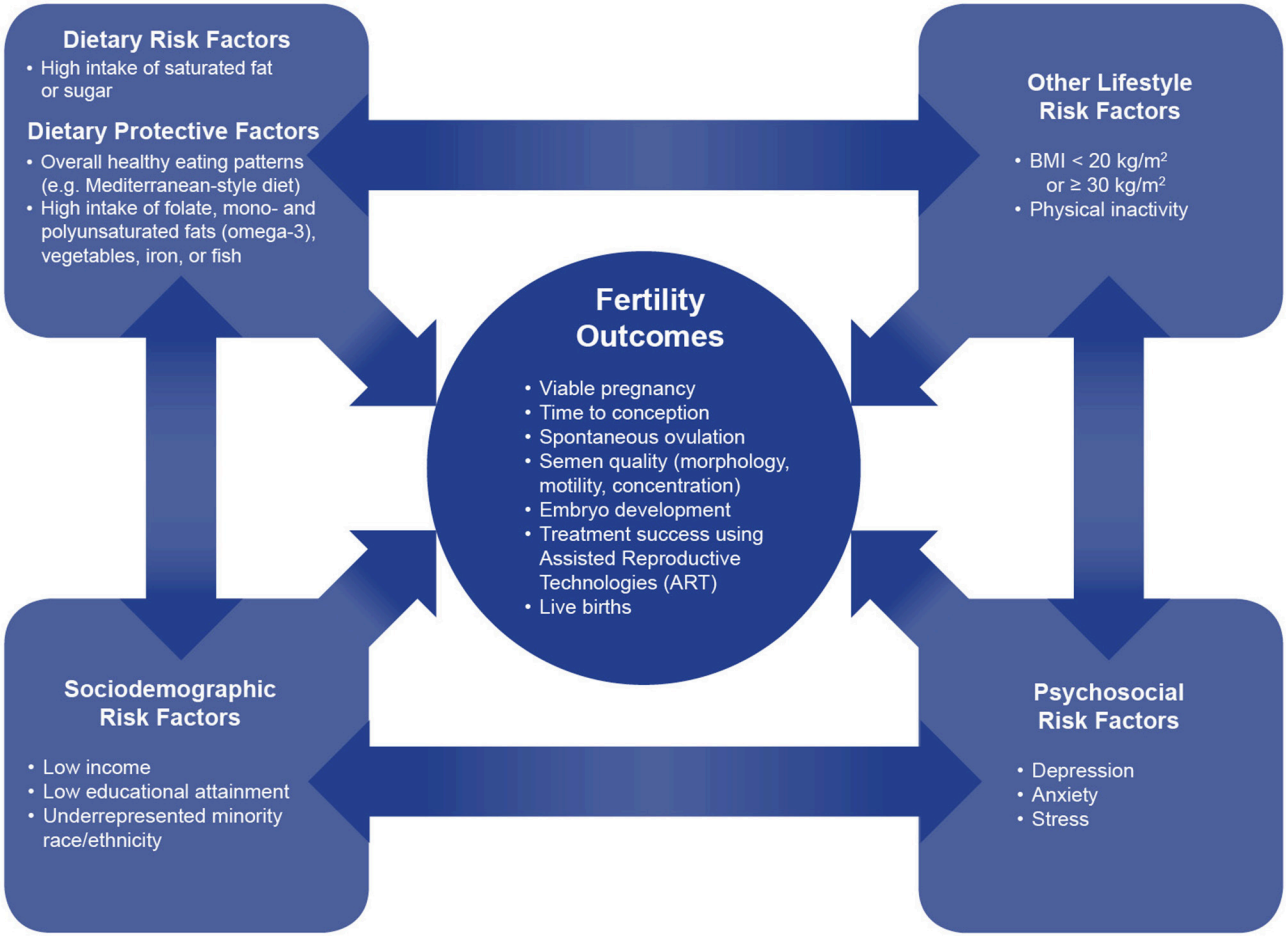
The interconnection between dietary, lifestyle, sociodemographic, and psychosocial factors on fertility outcomes. This conceptual framework shows the interconnections between dietary, lifestyle, sociodemographic, and psychosocial factors on fertility outcomes. Dietary factors, both protective and harmful, have bidirectional relationships with sociodemographic, psychosocial, and lifestyle risk factors. Dietary factors independently, as well as together with these correlated factors, impact multiple fertility outcomes.

The experience of infertility can exact a significant physical, psychosocial, and economic toll on couples (1, 2). An estimated 15% of couples in the United States (U.S.) are affected by infertility, defined as the failure to achieve pregnancy after 12 months of unprotected sexual intercourse (3). Although infertility is often associated with women, male physiological factors have been shown to be responsible for ~25% of cases, underscoring the need to consider both partners (4). The incidence of infertility has remained high despite increased use of assisted reproductive technologies (ART) in recent years (5). As such, research has aimed to identify modifiable risk factors for infertility; to date, nutritional factors have been the subject of much of this investigation. Evidence suggests that nutrition can play an important role in altering fertility-related outcomes in both men and women (5). The purpose of this article is to summarize the literature on the nutritional factors related to infertility and to consider the public health implications of this body of research within the landscape of the U.S (Figure 1).

## Evidence on diet and fertility

### Overall dietary patterns

There is strong evidence that healthy preconception dietary patterns among both men and women of reproductive age have a beneficial effect on fertility. A dietary pattern consistent with the recommendations put forth by the U.S. Dietary Guidelines for Americans, which recommends a high consumption of whole grains, monounsaturated or polyunsaturated oils, vegetables, fruits, and fish (6), has been associated with improved fertility in women and higher semen quality in men (5). In the Nurses' Health Study (NHS) II, a large prospective cohort, women who had the highest intake of a “fertility diet” comprised of plant protein from vegetable sources, full-fat dairy foods, iron, and monounsaturated fats, during the preconception period, were found to have a 66% (95% CI, 52, 77%) lower risk of infertility related to ovulatory disorders and a 27% (95% CI, 5, 43%) lower risk of infertility due to other causes compared to women with the lowest intake of this diet pattern, controlling for age, body mass index (BMI), alcohol intake, coffee intake, smoking, and oral contraceptive use (7). Population attributable risk calculations based on this sample suggest that not following the “fertility diet” was the attributable factor in 46% of cases of infertility, which was higher than all other independent risk factors (e.g., BMI, physical activity) (7). In another study of college-educated women in Spain, those in the highest quartile of adherence to a Mediterranean-style diet, which similarly included high intake of vegetables, fish, and polyunsaturated oils, had 44% (95% CI, 35, 95%) lower odds of seeking medical help for difficulty getting pregnant compared to women in the lowest quartile (8). The Mediterranean diet yielded similar benefits on achieving clinical pregnancy and live birth among non-obese women in Greece, but only for those below the age of 35 (9). Furthermore, data indicate that a healthy diet, consisting of the aforementioned food groups, improves measures of semen quality, including morphology, motility, and concentration (4).

### Specific foods and nutrients

Data on the associations of specific nutrients and foods with fertility may yield important insight into the possible mechanisms linking diet and reproductive health. In addition to being linked to neural tube defects in infants, low levels of folate are associated with a lower frequency of sporadic anovulation (10). In a randomized controlled trial of subfertile women who took a multivitamin containing 400 μg of folic acid for 3 months, 26% had a pregnancy compared to 10% of women in the placebo group (11). However, the dose-response benefit of folate appears to extend beyond the current recommended dose for reproductive-aged women (400 μg). Gaskins et al. (10) found that higher levels of pre-pregnancy folate supplementation were associated with a lower risk of spontaneous abortion, but only when comparing those who consumed greater than 730 μg per day of supplemental folate with those who did not consume any folate from supplements.

Preliminary data suggest that red meat may have an adverse effect on fertility. Results from an infertility cohort study showed that consumption of red meat was negatively associated (OR: 0.81; 95% CI, 0.65, 0.99) with likelihood of blastocyst formation during embryo development (12). Notably, iron intake may be reduced if red meat intake is restricted; yet it has been shown that consuming iron supplements and non-heme iron from other sources may decrease the risk of ovulatory infertility (13). Saturated fat content, which can be particularly high in red meat, has independently been linked to lower semen concentration in males (14). Polyunsaturated fats, conversely, have been shown to yield reproductive benefits in both men and women. A cross-sectional study of men showed that higher intake of omega-3 fatty acids was associated with significantly more favorable sperm morphology (14). Women who consumed higher levels of omega-6, linoleic acid, and omega-3 had a higher incidence of pregnancy than those with lower intake of these nutrients (15).

Current research examining the effect of dairy on fertility is limited in scope (5). One study found few and inconsistent associations between preconception dairy intake and fertility in two cohorts of reproductive-aged women (16). In the NHS II, however, while no relationship was found between total intake of dairy products and risk of infertility, full-fat dairy products were associated with a lower risk of ovulatory infertility while low-fat dairy products (including skim, 1%, and 2% milk, yogurt, or cottage cheese) were associated with a higher risk (17).

Studies have similarly yielded inconclusive evidence on the effect of alcohol and caffeine intake on fertility. Chavarro et al. (18) found that neither alcohol nor caffeine intake appeared to impair ovulation to the point of decreasing fertility in NHS. While men with caffeine intake greater than 272 mg/day and alcohol intake over 22 g/day have been linked to lower adjusted live birth rates after use of ART, intake of these substances has not been shown to affect semen quality (19). In another prospective cohort study of 3,628 women planning to become pregnant, women who reported consuming 3 or more servings of soda per day had a 52% lower (95% CI, 0.21, 1.13) rate of pregnancy compared to women who did not report any soda consumption, while there was no association found between coffee consumption and fertility (20). These results may be indicative of the adverse effects of sugar intake on fertility among women, although further studies on this topic are needed. Existing data suggest that high consumption of sugar is associated with lower semen quality and increased infertility among men (21).

### Body mass index

Current research indicates a roughly “J”-shaped relationship between BMI and fertility, such that the risk of infertility is highest among those at the lowest and highest ends of the BMI distribution (7). In the NHS II, compared to women classified as having recommended weight (BMI 20–25 kg/m^2^), a higher risk of ovulatory disorder infertility was observed for women classified as underweight (BMI < 20 kg/m^2^; RR: 1.38; 95% CI, 1.03, 1.85) and for women with obesity (BMI ≥ 30 kg/m^2^; RR: 2.35; 95% CI, 1.78, 3.11), after controlling for diet, age, smoking, and oral contraceptive use (7). Additionally, a review of the literature related to male obesity and fertility concluded that male obesity is associated with increased risk of infertility, potentially through endocrine dysregulation mechanisms (22). Obesity status has also been linked to ART treatment success. Among a nationally representative sample of U.S. women using ART, a BMI between 30 and 35 kg/m^2^ was associated with significantly greater odds (OR: 1.14; 95% CI, 1.09, 1.19) of failing to achieve a clinical intrauterine gestation compared to women in a reference group with BMI 18.5-25 kg/m^2^ (23).

There is limited data on the extent to which BMI modifies the relationship between dietary (and other) factors and infertility. Chavarro et al. (7) found that the relationship between an ideal dietary pattern and risk of infertility was not modified by BMI; although the absolute risk of ovulatory disorder infertility was higher in those with a BMI over 25 kg/m^2^ compared to those with a recommended BMI; the extent to which dietary improvements attenuated that risk was similar in both groups. Furthermore, physical activity levels also reduced the risk of ovulatory disorder infertility similarly across BMI categories (7).

A recent systematic review explored the impact of weight loss interventions among participants with overweight or obesity status on fertility-related outcomes (24). Among women, a pooled analysis of randomized studies found that participants randomized to active diet and exercise interventions were more likely (RR: 1.59; 95% CI, 1.01, 2.50) to become pregnant compared to control participants. While the literature was more sparse for men, one diet and exercise intervention in a cohort of subfertile men yielded significant improvements in the degree of sperm DNA fragmentation (24).

## Public health implications of the diet-fertility connection

### Integration of nutrition counseling into fertility treatment

Given the rigorous evidence presented above that suggests that various aspects of nutrition contribute to a reduced risk of fertility problems in the general reproductive-aged population and may also be an effective treatment for men and women already experiencing infertility, nutrition, and/or obesity counseling is likely to be central in fertility treatment. Obesity assessment is customary during the treatment process; 43% of U.S.-based infertility clinics included in a survey had a BMI cutoff for performing ART procedures, while 83% of the directors of clinics surveyed believed that a standard cutoff should exist (25). When asked about the weight loss method that they recommended to patients with an elevated BMI, 95% of respondents reported that they counseled their patients on proper diet and exercise, and 90% reported referring their patients to a nutritionist (25). This course of action is promising given the effectiveness of weight loss interventions on fertility outcomes (24). However, as noted above, evidence suggests that following a healthy diet provides a similar magnitude of benefit on fertility regardless of BMI status (7). Thus, it may be prudent to consider expanding weight-loss or nutritional advice to all individuals accessing infertility treatment, while continuing to prioritize those who are below or above certain BMI cutoffs. One way to support this would be to include nutritional counseling in national clinical guidelines for fertility. For example, the National Institute for Health and Care Excellence in the United Kingdom included in their 2013 fertility treatment guidelines that providers should inform patients experiencing difficulty becoming pregnant that either partner having a BMI > 30 kg/m^2^ may have a reduced chance of conception, and that losing weight might improve the chances of becoming pregnant (26). Nonetheless, fertility-promoting diets are not specifically mentioned in these clinical practice guidelines.

### Nutrition and the psychological burden of infertility

Women who experience issues with fertility are at an increased risk of depression relative to women not experiencing such problems (2). Moreover, women with pre-existing depression may be more likely to experience infertility due to physiological changes in hormone production and ovulation (27). A recent meta-analysis reported higher achievement of pregnancies or live births among women with lower pre-pregnancy depression or anxiety (28). Moreover, among men, exposure to occupational stressors was negatively associated with semen quality (29). As such, it is critical to understand how to manage depression and other psychosocial factors in women and men who are contemplating or having difficulty becoming pregnant. Beyond the effect of a healthy diet on fertility-related outcomes, certain dietary patterns have been shown to protect against depression. For example, participants randomized to a Mediterranean diet had a lower risk of depression compared to control participants who were assigned to a low-fat diet, especially among those with preexisting type 2 diabetes (30). Furthermore, the relationship between low folate status, a risk factor for subfertility, and depression has been well-characterized in the literature (31). Therefore, promoting healthy dietary patterns and higher folate intake among individuals experiencing infertility may improve their chances of achieving a pregnancy and concomitantly temper the psychological burden associated with their experience. However, current guidelines for psychosocial counseling in infertility treatment do not include nutritional advice as a factor that clinicians should consider (32).

### Consideration of fertility when developing nutritional guidelines

While several foods and nutrients that may protect against infertility are consistent with current federal nutrition guidelines—such as the USDA Dietary Guidelines for Americans (6)—the connection between diet and fertility is not mentioned. This omission may limit recommendations of foods and nutrients that have strong evidence for improved fertility at the population level. For example, women of reproductive age are recommended to consume 400 μg of folic acid per day, but evidence suggests that higher consumption pre-pregnancy may lower the risk of some infertility outcomes (10). Furthermore, while fish high in omega-3 fatty acid content are generally recommended as part of a healthy diet, there is potential for environmental contamination from mercury and other toxins in some specific type of fish. Although studies have reported mostly null associations between mercury intake and fertility or reproductive outcomes (33–36), specific recommendations for fish intake are made for pregnant women or women of childbearing age (37). Nonetheless, omega-3 fatty acid intake should still be recommended to these groups as part of a healthy fertility diet (38). Recognizing the types and quantities of foods that contribute to reproductive health may improve national nutrition guidelines.

### Nutrition and sociodemographic disparities in infertility

In the U.S., women with lower income or lower educational attainment experience a higher prevalence of infertility outcomes compared to those with higher income or educational attainment, while Hispanic and non-Hispanic Black women have a higher prevalence of infertility compared to non-Hispanic white women (1, 39). The disparity in fertility rates may partially be explained by nutritional intake, as recent data from a large cohort (*n* = 7,511) of nulliparous women showed that in the months prior to conception, women with lower educational attainment or who were Hispanic or non-Hispanic Black, had a poorer general diet than women with higher educational attainment or who were non-Hispanic White (40). Disparities have also been reported in receipt of preventive services for optimal diet and health for conception among U.S. women and men of reproductive age (41). These results reflect the current state in the general U.S. population, as groups who are racial/ethnic minorities or have lower education tend to have poorer diet quality as well as a higher prevalence of obesity, which is another risk factor for infertility (42).

## Conclusions and recommendations

This mini-review summarizes existing evidence on the relationship between fertility and nutrition. While there is a well-characterized association between high intake of folic acid, polyunsaturated fats, and plant-based foods on fertility outcomes, further research is required to more clearly understand the roles of other foods. Future research should also consider the need for randomized controlled trials and studies examining the combined effects of diets of both male and female partners on fertility. Despite recent progress in the amount of literature on the relationship between fertility and diet, this is the first article, to our knowledge, to specifically focus on the public health implications of this connection; a few articles have briefly and partly discussed the topic (5, 10). Thus, there is a need for more standardized integration of nutrition counseling into treatment delivery for infertility. Incorporating specific guidelines for individuals of reproductive age when developing federal nutritional guidelines may also impact the reach of this information. Furthermore, there is an urgent need to develop targeted messaging and interventions among individuals with extreme BMI categories, racial/ethnic minorities, and low-income and low-education groups in order to improve existing disparities in both fertility and overall health outcomes in the U.S.

Given the positive effect of a healthy diet on fertility outcomes, and the implications for public health and clinical practice noted above, several recommendations can be noted. First, clinicians should provide counseling to improve dietary behaviors among patients accessing ART services, regardless of BMI while prioritizing those with unfavorable BMI. Specifically, a diet consistent with the U.S. Dietary Guidelines for Americans and adequate levels of folic acid intake for women could be recommended. Moreover, patients with a BMI consistent with underweight, overweight, or obesity status should be referred to nutritional and weight-loss counseling to help improve the likelihood of positive fertility outcomes. In order for health care providers to have the best and most current information on the effects of specific dietary components on fertility, there should be clear and effective communication between researchers and clinicians.

A second recommendation is to improve the delivery of nutritional programs and interventions aimed at the general reproductive-aged population, as well as targeted at-risk groups. For example, considering accessibility of an intervention has been cited as an important factor contributing to young adults' willingness to participate in a weight loss program (43). Thus, leveraging the ubiquity of mobile technology, such as text messaging or a Smartphone application, to deliver a healthy eating or weight loss intervention has been shown to be an effective strategy to engage this sector of the population (44). Given the sociodemographic and racial/ethnic disparities in infertility, efforts to improve the diets of reproductive-aged adults should target low socioeconomic or education groups and racial/ethnic minority populations. Interventions should consider the unique barriers these groups face related to optimal nutrition, including structural barriers such as access to healthy foods in terms of geographic accessibility as well as price (45, 46). Approaches that considers the structural barriers to a healthy diet among this target population may involve limiting the extent to which the U.S. government-assisted food benefit program Supplemental Nutrition Assistance Program can be used to purchase foods that contribute to infertility, such as sugary beverages, or incentivizing the purchase of foods consistent with a healthy diet pattern for reproductive-aged adults through a program similar to the Healthy Incentive Program (47).

In order to lend further support to fertility-promoting diets for the population, a third recommendation is to include evidence-based messaging in population-wide nutritional guidelines such as the U.S. Dietary Guidelines for Americans. The public health implications of nutrition and fertility in the U.S. are multifaceted. Future research and collaboration across stakeholders in research institutions, clinical practice, and the community will help drive and implement more evidence-based recommendations and interventions.

## Author contributions

NP and AG researched and analyzed the background literature, and wrote the manuscript, including interpretations. MT and JM conceptualized the topic, provided substantial scholarly guidance on the manuscript draft and interpretation, and revised the manuscript critically for intellectual content. All authors approve the final version of the manuscript, ensure the accuracy and integrity of the work, and agree to be accountable for all aspects of the work.

### Conflict of interest statement

The authors declare that the research was conducted in the absence of any commercial or financial relationships that could be construed as a potential conflict of interest.
